# A clinical case report of brain abscess caused by *Nocardia brasiliensis* in a non-immunocompromised patient and a relevant literature review

**DOI:** 10.1186/s12879-020-05052-0

**Published:** 2020-05-07

**Authors:** Jian-Wei Zhu, Hui Zhou, Wei-Qiang Jia, Jian You, Ru-Xiang Xu

**Affiliations:** 1 Department of Neurosurgery, Sichuan Provincial People’s Hospital, University of Electronic Science and Technology of China, Chengdu, 611731 China; 2 Department of Neonatology, Traditional Chinese Medicine Hospital of Southwest Medical University, Luzhou, China; 3 Department of Neurosurgery, The Affiliated Hospital of Southwest Medical University, Luzhou, China

**Keywords:** Brain abscess, *Nocardia brasiliensis*, Non-immunocompromised, Surgical treatment, Effective antibiotics, Recurrence

## Abstract

**Background:**

Brain abscess due to the *Nocardia* genus is rare and usually found in immunocompromised patients. The most common subtype implicated is *Nocardia farcinica* while brain abscess due to *Nocardia brasiliensis* is comparatively rare. Diagnosis of brain abscess is based mainly on bacteriological culture from pus collected at the site of infection, and brain imaging. Stereotaxic aspiration or surgical resection combined with adequate duration of treatment with antibiotics to which the bacteria are sensitive represent effective treatment strategies.

**Case presentation:**

We report a rare case of brain abscess caused by *Nocardia brasiliensis* in a non-immunocompromised patient. He admitted to our hospital twice with a headache. Stereotaxic aspiration was performed at the patient’s first appointment at the hospital, and a craniotomy was used to excise the lesion during subsequent abscess recurrence.

**Conclusion:**

Early diagnosis, reasonable surgical intervention, and adequate duration of treatment with effective antibiotics are critical for treating brain abscess.

## Background

Brain abscess caused by infectious disease is a common and severe intracranial condition. In previous decades, it was associated with high incidence, disability, and mortality rates [1], owing to the lack of effective diagnostic and treatment strategies. With advancements in imaging technologies, the morbidity and mortality rates associated with brain abscess have reduced in recent decades [2]. Pathogenic bacteria found in a brain abscess enter the brain tissue through hematogenous spread, closer spread (e.g., ear infection) and traumatic infection in patients with head trauma or surgery.

Nocardial brain abscess is rare and typically found in immunocompromised patients [3]. *Nocardia* species are aerobic, gram-positive, branching, and filamentous bacteria, and can be introduced into a host through inhalation. *Nocardia* infections comprise only 2% of all intracranial abscesses [4], but the mortality rate (31%) is greater than that for other types of infections (< 10%). *Nocardia farcinica* species are responsible for more than 80% of nocardial brain abscesses, and brain abscess caused by *Nocardia brasiliensis* species is rarely reported. Here, we report a brain abscess caused by *N.brasiliensis* infection in a patient without known risk factors. The publication of this case report obtained written informed consent from the patient.

## Case presentation

**Fig. 1:**
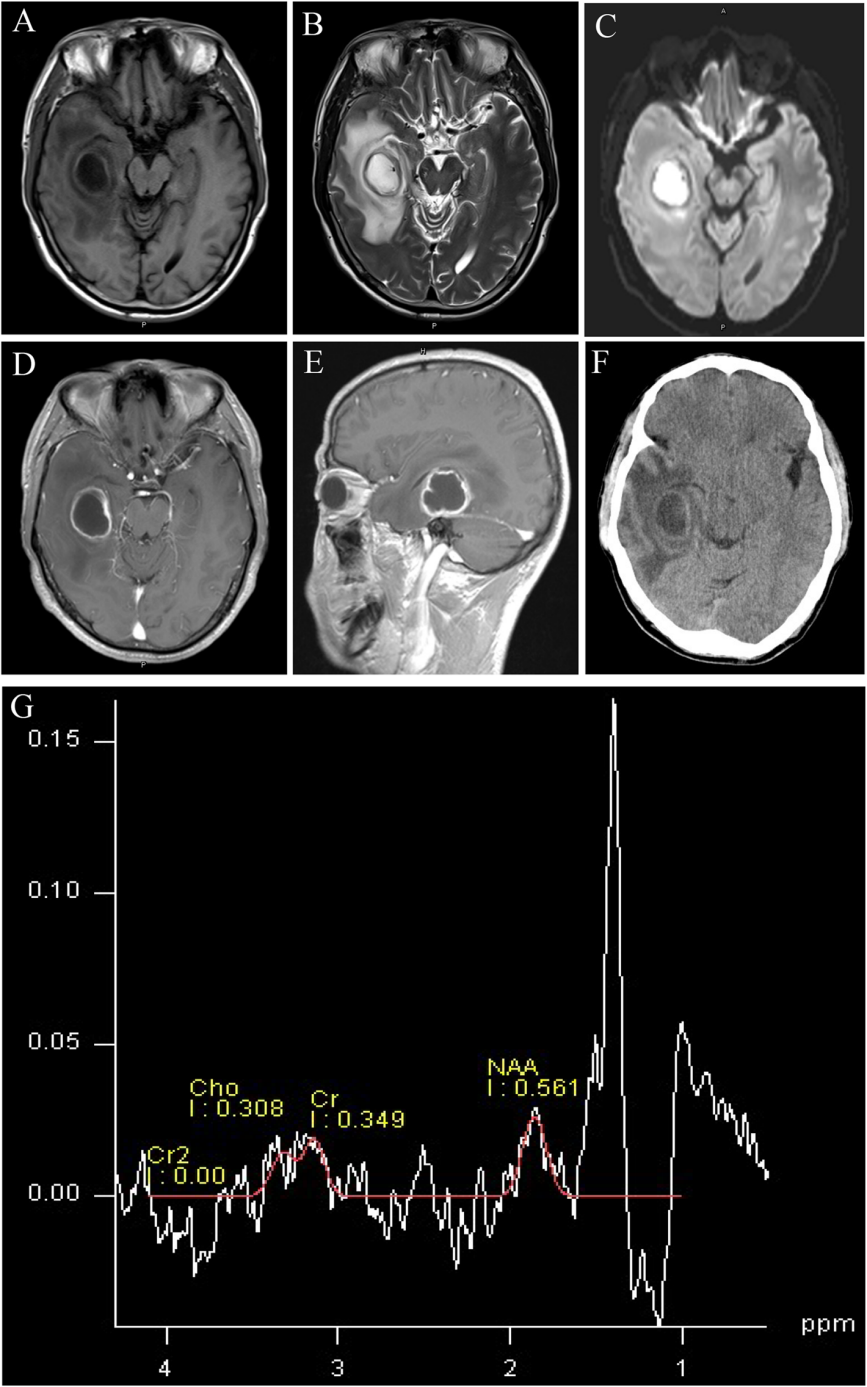
Head MRI and CT images prior to the first surgery. **a-b** A quasi-circular space-occupying lesion was found with long T1-weighted and T2-weighted signal shadows in the right temporal lobe and was surrounded by large patches of edema. **c** Enhanced T1-weighted FLAIR signal were observed in the center of lesion. **d-e** The signal intensity of the ring wall was increased after enhancement. **f** CT scan showed a circular space-occupying lesion with slightly high density ring wall and low density shadows in the right temporal lobe that was surrounded by large patches of edema. **g** MRS showed an elevated lactic acid peak, and low NAA, choline, and creatine peaks

A male aged 52 years was admitted to our outpatient department with a headache. His headache first appeared one month prior to admission to our hospital, and worsened severely at six hours prior to admission. He had a history of good physical health (without organ transplants, leukemia, diabetes, underlying malignancy, human immunodeficiency virus, long-term use of steroid and autoimmune disease) and did not report any foci of infection, sinusitis, or head trauma. The patient was hospitalized with severe headache as an inpatient in our department. Physical examination showed that the patient was conscious with a Glasgow Coma Scale score of 15, had sensitivity to light reflex, and a soft neck. He reported no vomiting or blurred vision. The patient reported muscle weakness in the left lower limb (4/5). His body temperature was 36.7 °C. His complete blood count indicated a white blood cell count of 11.4 × 10^9^/L, 7.5% lymphocytes, 88.3% neutrophils and a C-reactive protein level of 1.1 mg/L. Head magnetic resonance imaging (MRI) showed a sharply defined lesion (3.1 cm × 2.4 cm × 2.9 cm) in the right temporal lobe with long T1-weighted and T2-weighted signals shadows and a circular equisignal (Fig. 1a, b). Enhanced diffusion-weighted imaging signals were observed in the center of lesion (Fig. 1c). The signal intensity of the ring wall was increased after enhancement, and the thickness of the ring wall was uniform (Fig. 1d, e). The lesion was surrounded by large patches of edema (Fig. 1b; CT scan image in Fig. 1f). The adjacent lateral ventricles and parenchyma displayed compression deformation and the midline structures were slightly shifted toward the left (Fig. 1). Magnetic resonance spectrum (MRS) showed an elevated lactic acid peak, and low N-acetyl aspartate (NAA), choline, and creatine peaks (Fig. 1g).

**Fig. 2:**
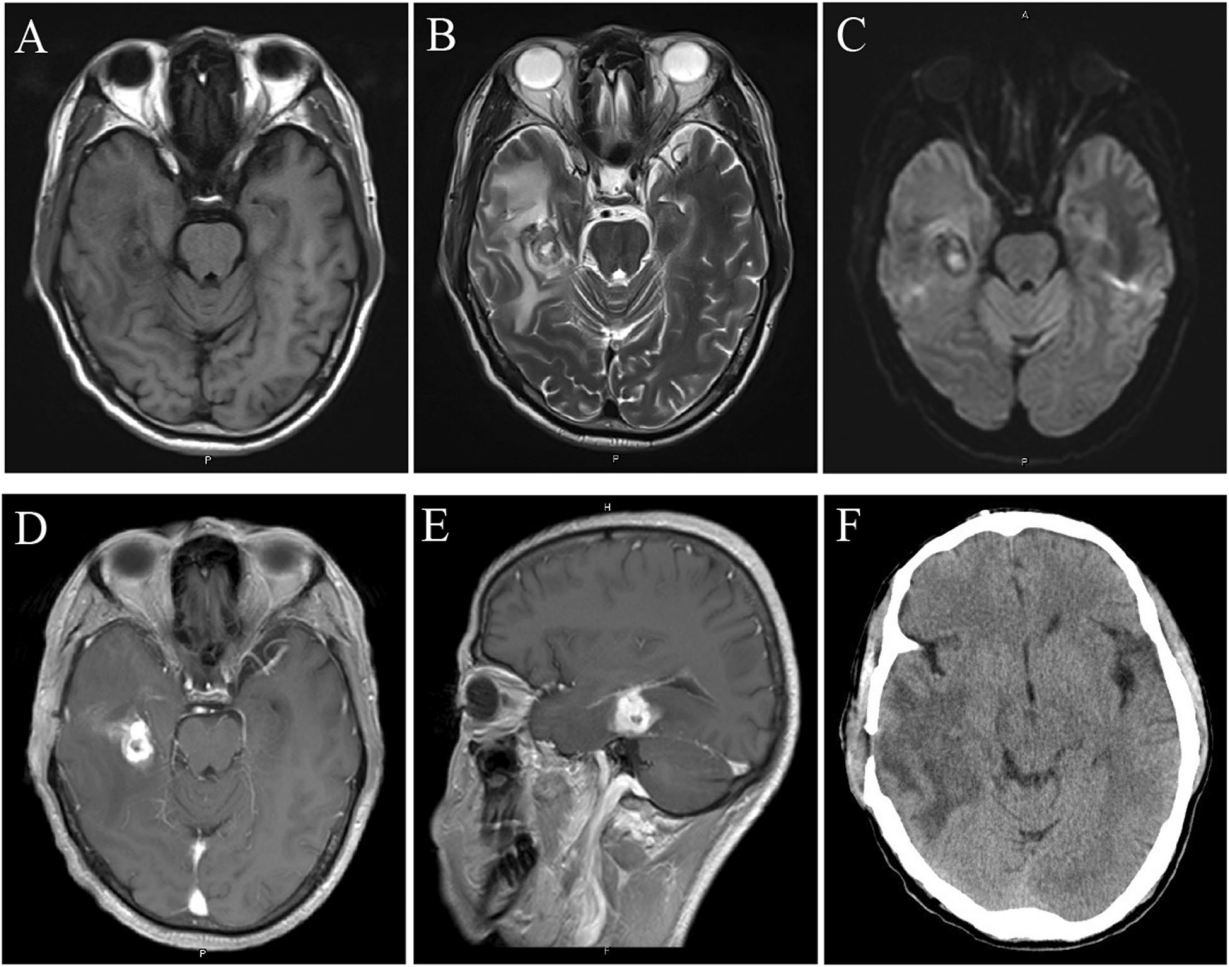
Head MRI and CT images after the first surgery. **a-b** The lesion disappeared but the wall of the abscess remained, with slightly long T1-weitghted and long T2-weighted signal shadows surrounded by large patches of edema. **c** T2-weighted FLAIR signal showed a slightly enhanced lesion with a low signal ring wall that was surrounded by patches of edema. **d-e** The signal intensity of the ring wall was increased after enhancement. **f** CT scan showed that the lesion has disappeared, but the large patches of edema were still present

After diagnosis of brain abscess based on the imaging results, puncture aspiration of the space-occupying lesion in the right temporal lobe was performed. Dark yellow pus was extracted during the operation, but the capsule (abscess wall) was left untreated. The patient’s headache was alleviated, but he had a low-grade fever that fluctuated around 37.3 °C after the operation. The patient was initially treated with meropenem (1 g intravenous (i.v.) every 8 h) according to standard practice. Pus culture showed Gram positive, partially acid-fast, and strictly aerobic filamentous bacilli and was further identified as *N. brasiliensis* by 16S ribosomal RNA (rRNA) sequencing after 3 days cultured. 16S rRNA gene sequencing revealed 99% homology with that of strain *N. brasiliensis* at the GenBank database. A drug sensitivity test (E-test method) showed that the bacterium was sensitive to amikacin, ceftriaxone, trimethoprim, gentamycin, doxycycline, linezolid, piperacillin–tazobactum, meropenem. The patient was then treated with amikacin (2 g i.v. every 12 h) and ceftriaxone (2 g i.v. every 12 h). After 10 days, the patient’s temperature returned to normal and his headache completely disappeared. The complete blood count results were also normal. The head MRI showed that the space-occupying lesion in the right temporal lobe had disappeared (Fig. 2a-c) and the patches of low-density shadows were substantially reduced (Fig. 2b; CT scan image in Fig. 2f). The midline structures were no longer shifted, but the wall of the abscess was still apparent (Fig. 2d, e). Physical examination showed that the patient was conscious, and had normal limb muscle strength and normal body temperature. As such, we agreed to the discharge the patient in accordance with his request.

**Fig. 3:**
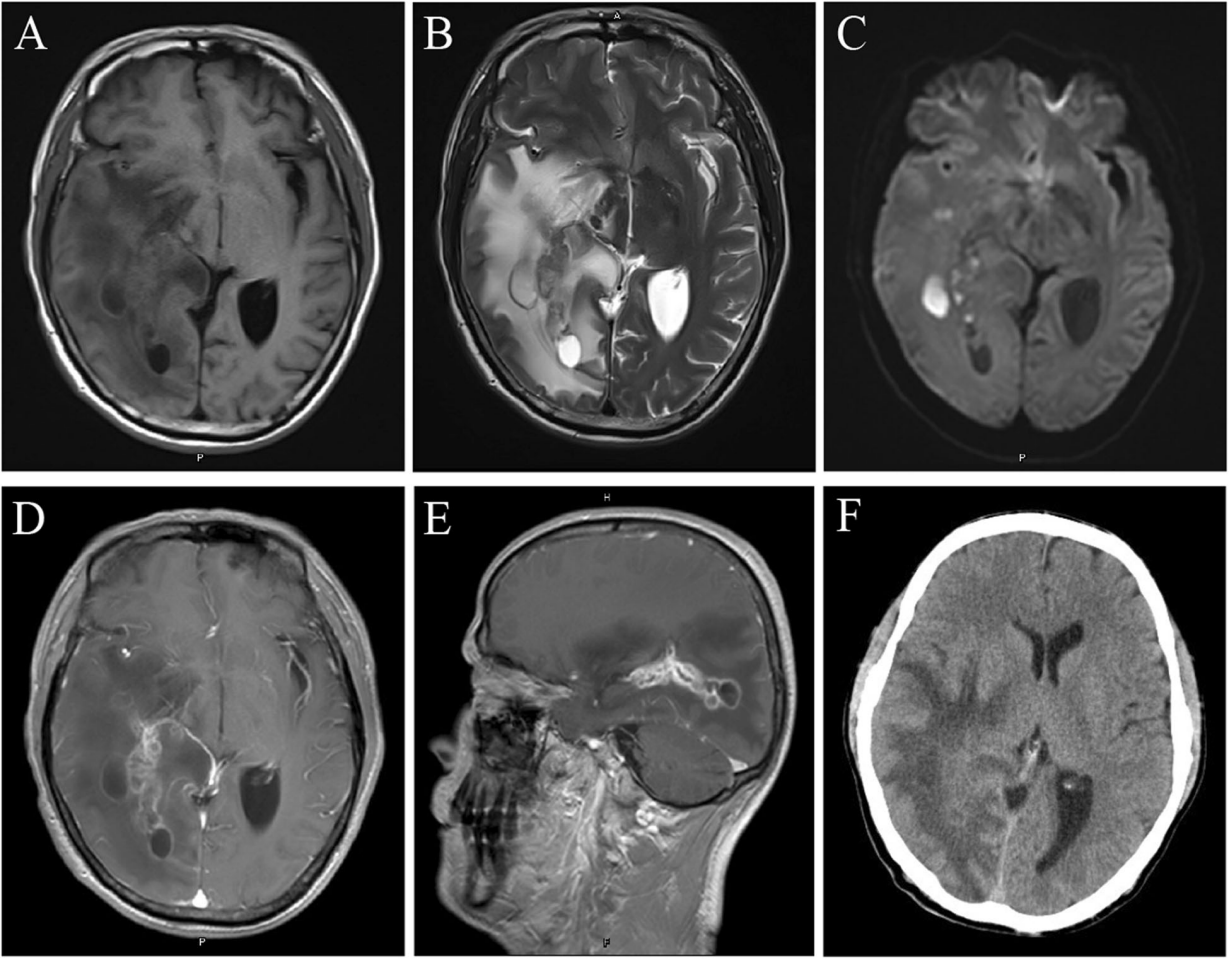
Head MRI and CT images before the second surgery. **a-b** Multiple irregular space-occupying lesions were found with slightly long T1-weighted and T2-weighted signal shadows in the right temporal lobe and occipital lobe that were surrounded by large patches of edema. **c** T2-weighted FLAIR showed high-intensity signals. **d-e** The signal intensity of the lesion ring wall of lesions was slightly increased after enhancement. **f** CT scan showed irregular low density edema shadows in the right temporal lobe and occipital lobe

However, the patient was readmitted to our hospital three months after discharge with severe headache. The headache had progressively worsened over the previous 10 days, and was associated with vomiting. Physical examination showed that the patient was conscious with a Glasgow Coma Scale score of 15, exhibited sensitivity to light reflex, a soft neck, and normal limb muscle strength. Body temperature was 36.8 °C, and he had a white blood cell count of 10.4 × 10^9^/L, 10.5% lymphocytes, 83.5% neutrophils, and a C-reactive protein level of 0.5 mg/L. Head MRI showed multiple irregular lesions (the maximum lesion measured 2.1 cm × 1.4 cm × 0.8 cm) in the right temporal lobe and occipital lobe with slightly long T1-weighted and T2-weighted signals shadows (Fig. 3a, b) and equi- or low- intensity fluid-attenuated inversion recovery (FLAIR) signals were observed (Fig. 3c). The signal intensity of the ring wall was slightly increased after enhancement (Fig. 3d, e) and the lesion was surrounded by large patches of edema (Fig. 3b; CT scan image in Fig. 3f). The adjacent lateral ventricles and parenchyma displayed compression deformation and the midline structures were slightly shifted toward the left (Fig. 3).

**Fig. 4:**
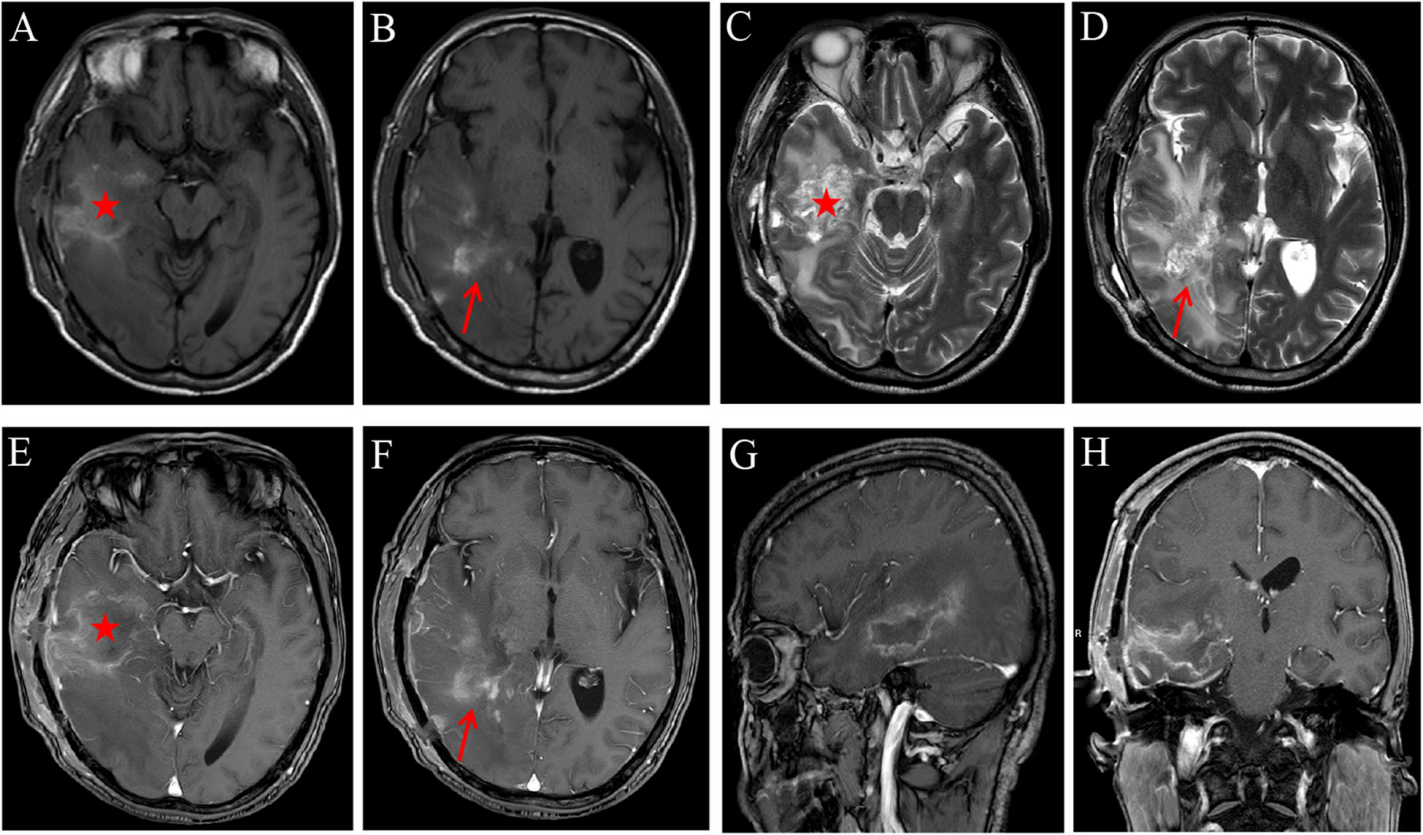
Head MRI images after the second surgery. **a-d** Plaque-like long T1-weighted and T2-weighted signal shadows in the right temporal lobe and occipital lobe. **e-h** No substantially enhanced signal after enhancement. Asterisk = the area of the abscess in the first admission. Arrow = the area of the recurrent abscess

**Fig. 5:**
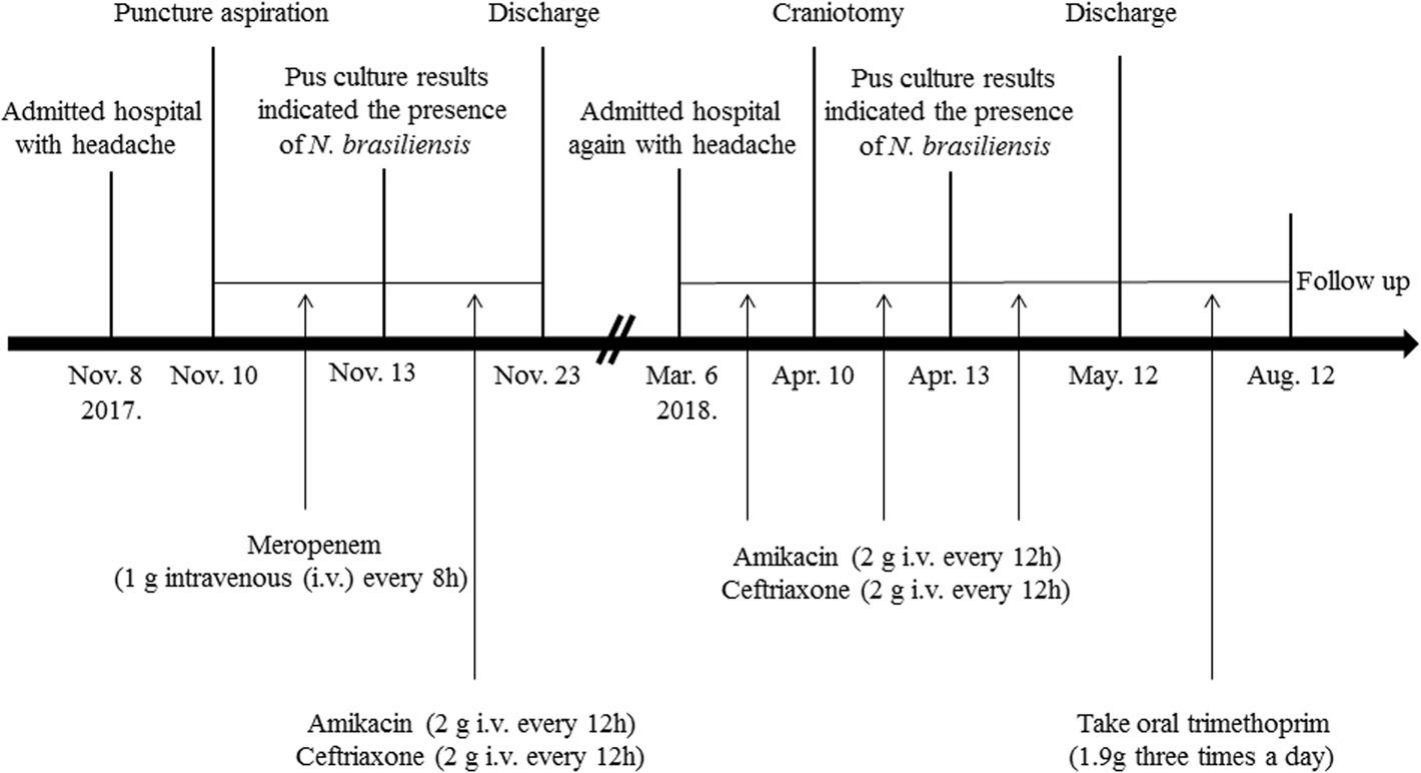
Timeline of case presentation description

To excise the lesion, we performed craniotomy; yellow pus was extracted during the operation and the capsule (abscess wall) was not visible. The patient’s headache was alleviated; however, he again had a low-grade fever that fluctuated around 37.5 °C after operation. The patient was treated with amikacin (2 g i.v. every 12 h) and ceftriaxone (2 g i.v. every 12 h) according to his previous postoperative therapy. Pus culture results again indicated the presence of *N. brasiliensis*, and it was also sensitive to amikacin, ceftriaxone, trimethoprim, gentamycin, doxycycline, linezolid, piperacillin–tazobactum, meropenem. Patient continued to be treated with amikacin (2 g i.v. every 12 h) and ceftriaxone (2 g i.v. every 12 h). After seven days, his temperature returned to normal and the headache completely disappeared. The complete blood count results were also normal. The head MRI showed that the space-occupying lesions in the right temporal lobe and occipital lobe had disappeared (Fig. 4) and the patches of low-density shadows were substantially reduced (Fig. 4c, d). The midline structures were no longer shifted (Fig. 4). Physical examination showed that the patient was conscious, and had normal limb muscle strength. The patient continued to receive treatment with amikacin (2 g i.v. every 12 h) and ceftriaxone (2 g i.v. every 12 h) for three weeks before discharge. Additionally, the patient continued to take oral trimethoprim (1.9 g three times a day; the dose was calculated by weight) for three months after discharge. There was no recurrence at the time of the one-year postoperative follow up (Fig. 5).

## Discussion and conclusions

*Nocardia* affects the lungs more frequently than the central nervous system [5]; infections account for 2% of brain abscesses, and usually affect immunocompromised patients, such as those with HIV co-infection and those receiving cancer chemotherapy or long-term immunomodulatory therapy [4, 6, 7]. Infection due to *N. brasiliensis* is rare compared with that due to other species, which generally results from direct traumatic inoculation into the skin and hematogenous spread from the primary location to the central nervous system where a brain abscess is formed. However, rare cases of meningitis or spinal abscess caused by *N. brasiliensis* have also been reported [8].

The clinical course of brain abscess caused by *Nocardia* is typically gradual, with symptoms presenting over months or even years. Imaging evidence contributes to identifying brain abscesses in the cases of tumors and cystic or necrotic foci. The abscess usually develops a vascularized wall composed of astrocytes and glia that can be identified by CT scan or MRI as a ring enhancement depending on the capsular phase [9]. During the development of brain abscess, three stages of infections (meningoencephalitis stage; suppuration, and capsule stages) can be distinguished on the basis of evidence from brain imaging [10, 11]. The cranial CT scan and MRI data from the patient’s first hospital visit both revealed a ring enhancement. However, this ring enhancement was not obvious on the scans of his recurrent abscess. The reason for the abscess of the ring enhancement in subsequent scans might be that the abscess and edema formed relatively slowly, and there was some compensation for elevated cranial pressure during this process. If the patient’s headache appeared at a comparatively later stage, there would have been enough time for the lesion to form a vascularized before the patient visited the hospital for the first time. However, it is easier for the brain tissue with prior vasogenic edema to form edema again; further, the compensation for elevated cranial pressure is relatively weak, both of which may have led to earlier presentation of the headache, explaining why patient presented at the hospital sooner the second time.

In the capsule stage, brain abscesses are similar to gliomas and cystic or necrotic foci on imaging modalities and require further tests for differential diagnosis. MRS is a useful method for distinguishing between brain abscess, gliomas, and cystic or necrotic foci [10] . The center of a brain abscess lacks the metabolites of normal brain tissue; therefore, the peaks of NAA, creatine, and choline are low but the level of lactic acid is elevated. In addition, the signals of valine, leucine, and isoleucine are important markers for diagnosing brain abscess; however, the absence of the three amino acids does not exclude the presence of brain abscess [12, 13]. In our report, MRS displayed an elevated lactic acid peak and low NAA, creatine, and choline peaks. Diffusion-weighted imaging is also a valuable method to distinguish between brain abscess, brain glioma, and brain metastases. The abscess cavity manifests as a hyper-intense signal on T1-weighted FLAIR and has a low apparent diffusion coefficient (ADC). By contrast, a cystic or necrotic area of a brain tumor usually displays hypo-intense signals on T1-weighted FLAIR and high ADC values [2]. In our report, the hyper-intense signal on T1-weighted FLAIR and a low ADC values were found in the first time scan, but were not apparent in the recurrent brain abscess scans. We presume that this was because the abscess development had not fully reached the capsule stage in the recurrent episode.

Brain abscess diagnosis is mainly based on bacteriological cultures from pus [14]. In our case, we punctured aspirated pus and relieved cranial pressure. Bacteriological cultures indicated *N. brasiliensis* infection that was sensitive to amikacin and ceftriaxone. We continued to administer amikacin and ceftriaxone treatment for10 days after surgery the patient was discharged. Standard medical treatment for *Nocardia* brain abscess is trimethoprim/sulfamethoxazole; accordingly, we prescribed oral trimethoprim to the patient for three months. However, three months after discharge, the abscess recurred and the patient returned to the hospital.

Surgical treatment options for brain abscess include craniotomy excision and puncture aspiration. Craniotomy for excision of the entire abscess and capsule is more effective than aspiration. During our patient’s first admission to the hospital, we performed puncture aspiration surgery to treat the abscess, but the capsule wall was left. Retention of the capsule is an important factor in recurrence, when we performed a craniotomy to fully clear both the abscess and capsule at the patient’s second admission to the hospital, there was no further recurrence at one year of follow up. We found that the abscess capsule was located near the original lesion and there were multiple additional lesions. Multiple abscesses are found in 38% of patients with brain abscess due to *Nocardia* infection [15]. In our case, the abscess was single in his first admission to hospital. It is also possible that treatment time with intravenous antibiotics was inadequate after the first surgery and contributed to recurrence. Patients with brain abscess should receive intravenous antibiotics for 4–6 weeks, and administration of antibiotics should be maintained until the patient’s postoperative body temperature has remained within a normal rang for 10–14 days [10]. In the present case, the patient received only 10 days of intravenous antibiotics owing to his strong desire to be discharged. It should be noted that, for an immunosuppressed patient, 6–12 months of antibiotic treatment is required to prevent recurrence and increased risk of mortality [16]. After his second surgery to treat the brain abscess recurrence, our non-immunocompromised patient had received intravenous antibiotics for four weeks and continued to take oral trimethoprim for three months. At the time of the postoperative follow up at one year, there was no further recurrence.

In conclusion, brain abscess caused by *N. brasiliensis* in non-immunocompromised individuals is rare. In the present case, craniotomy for abscess excision was a more effective surgical method than aspiration. Complete excision of the abscess capsule was an efficient precaution against recurrence. It is necessary to choose antibiotics according to drug sensitivity tests results, and to ensure adequate duration of intravenous treatment for 4–6 weeks and until postoperative body temperature has remained within a normal rang level for 10–14 days. It is also essential for the patient to continue to take oral antibiotics (trimethoprim/sulfamethoxazole) for at least 3 months after discharge.

## Data Availability

Data sharing is not applicable to this article as no datasets were generated or analyzed during the current study.

## Abbreviations

MRI: Magnetic resonance imaging
MRS: Magnetic resonance spectrum
NAA: N-acetyl aspartate
FLAIR: Fluid-attenuated inversion recovery
ADC: Apparent diffusion coefficient
